# Novel prosurvival function of Yip1A in human cervical cancer cells: constitutive activation of the IRE1 and PERK pathways of the unfolded protein response

**DOI:** 10.1038/cddis.2017.147

**Published:** 2017-03-30

**Authors:** Yuki Taguchi, Yuta Horiuchi, Fumi Kano, Masayuki Murata

**Affiliations:** 1 Laboratory of Frontier Image Analysis, Graduate School of Arts and Sciences, The University of Tokyo, 3-8-1 Komaba, Meguro-ku, Tokyo 153-8902, Japan; 2 Department of Life Sciences, Graduate School of Arts and Sciences, The University of Tokyo, 3-8-1 Komaba, Meguro-ku, Tokyo 153-8902, Japan; 3 Cell Biology Unit, Institute of Innovative Research, Tokyo Institute of Technology, 4259 Nagatsuta, Midori-ku, Yokohama 226-8503, Japan

## Abstract

Cancer cells are under chronic endoplasmic reticulum (ER) stress due to hypoxia, low levels of nutrients, and a high metabolic demand for proliferation. To survive, they constitutively activate the unfolded protein response (UPR). The inositol-requiring protein 1 (IRE1) and protein kinase RNA-like ER kinase (PERK) signaling branches of the UPR have been shown to have cytoprotective roles in cancer cells. UPR-induced autophagy is another prosurvival strategy of cancer cells, possibly to remove misfolded proteins and supply nutrients. However, the mechanisms by which cancer cells exploit the UPR and autophagy machinery to promote survival and the molecules that are essential for these processes remain to be elucidated. Recently, a multipass membrane protein, Yip1A, was shown to function in the activation of IRE1 and in UPR-induced autophagy. In the present study, we explored the possible role of Yip1A in activation of the UPR by cancer cells for their survival, and found that depletion of Yip1A by RNA interference (RNAi) induced apoptotic cell death in HeLa and CaSki cervical cancer cells. Intriguingly, Yip1A was found to activate the IRE1 and PERK pathways of the UPR constitutively in HeLa and CaSki cells. Yip1A mediated the phosphorylation of IRE1 and also engaged in the transcription of PERK. The activation of these signaling pathways upregulated the expression of anti-apoptotic proteins and autophagy-related proteins. These events might enhance resistance to apoptosis and promote cytoprotective autophagy in HeLa and CaSki cells. The present study is the first to uncover a key prosurvival modulator, Yip1A, which coordinates IRE1 signaling with PERK signaling to support the survival of HeLa and CaSki cervical cancer cells.

Cancer cells are exposed continuously to a stressful microenvironment, for example, hypoxia and nutrient deprivation. They also have a high metabolic demand for growth, and these conditions cause chronic endoplasmic reticulum (ER) stress.^1, 2, 3, 4^ To cope with these harsh conditions, cancer cells activate a series of signaling pathways called the unfolded protein response (UPR), which promotes the recovery of ER function, as a prosurvival strategy.^1, 2, 3, 4^ Although activation of the UPR alleviates ER stress, under prolonged or severe ER stress, it leads to apoptosis to eliminate the stressed cells.^5, 6^ Cancer cells somehow modulate the signaling pathways, and constitutively activate the UPR without triggering apoptosis.

Recent studies have revealed that the branches of the UPR that involve inositol-requiring enzyme 1 (IRE1, also known as endoplasmic reticulum to nucleus signaling 1 (ERN1)) and protein kinase RNA-like ER kinase (PERK, also known as eukaryotic translation initiation factor 2-alpha kinase 3 (EIF2AK3)) have cytoprotective roles in cancer development and progression.^7, 8^ In response to ER stress, both IRE1 and PERK oligomerize and undergo trans-autophosphorylation.^9, 10^ The resulting activated IRE1 removes a short intron from X-box-binding protein 1 (XBP1) mRNA to yield spliced-XBP1 protein.^11^ Spliced-XBP1 activates the transcription of genes that function in ER-associated protein degradation (ERAD) and protein folding, resulting in the clearance of unfolded proteins from the ER and improved cell survival.^11, 12^ Despite the promotion of survival by IRE1-XBP1 signaling, recent studies have demonstrated that inhibitors of IRE1 endonuclease activity fail to sensitize cells to ER stress-induced apoptosis.^13, 14^ It is plausible that distinct signaling pathways downstream of IRE1 might promote cancer cell survival. In recent work, Hu *et al.*^15^ reported that IRE1 interacts with I*κ*B kinase (IKK) through TNF receptor-associated factor 2 (TRAF2) under ER stress, and thus activates nuclear factor kappa B (NF-*κ*B). In response to ER stress, the IRE1/TRAF2 complex phosphorylates IKK, which in turn phosphorylates inhibitor of *κ*B (I*κ*B) and promotes its degradation. Phosphorylated IKK also phosphorylates NF-*κ*B p65, a subunit of the NF-*κ*B complex, and leads to translocation of this prosurvival transcription factor into the nucleus. NF-*κ*B is a key transcription factor that regulates multiple processes of carcinogenesis, and constitutive activation of NF-*κ*B has been associated with various cancers.^16, 17^ The IRE1/TRAF2 complex was also reported to phosphorylate c-Jun N-terminal kinase (JNK) in response to ER stress.^18^ JNK is a member of the mitogen-activated protein kinase family and mediates prosurvival and proapoptotic signaling in cancer cells.^19^ Recently, it was demonstrated that the IRE1–TRAF2–JNK pathway contributes to ER stress-induced autophagy^20^ and that the activation of JNK coincides with the expression of several anti-apoptotic proteins early in the UPR.^21^ However, the molecular mechanisms by which ER stress activates the IRE1–IKK–NF-*κ*B and IRE1–JNK pathways during carcinogenesis remain to be elucidated.

Under ER stress, the PERK pathway also promotes cell survival.^10, 22^ Activated PERK phosphorylates eukaryotic translation initiation factor 2*α* (eIF2*α*), which attenuates global protein synthesis and decreases protein flux into the ER. Paradoxically, phosphorylated eIF2*α* enhances the translation of activating transcription factor-4 (ATF4). ATF4 translocates into the nucleus, where it upregulates UPR target genes required for autophagy, antioxidant response, and amino acid metabolism.^23, 24, 25^ UPR-induced autophagy is another prosurvival strategy of cancer cells.^26, 27, 28^ Autophagy is a catabolic process in which unwanted proteins are sequestered into autophagosomes and then degraded by lysosomal proteases.^29^ Autophagy has an important role under the UPR in maintaining ER homeostasis and supplying rapidly proliferating cancer cells with nutrients.^20, 30, 31^ However, it is currently unclear which branch of the UPR activates autophagy under ER stress.

In cancer cells, both the UPR and autophagy appear to protect the cells from apoptosis and promote cell survival. Molecules that mediate the cross talk between the two processes can be good therapeutic targets for cancer. Recently, we demonstrated that Ypt-interacting protein 1A (Yip1A, also known as Yip1 domain family member 5 (YIPF5)) mediates functional interconnection between the UPR and autophagy.^32^ Yip1A has been implicated in trafficking steps between the ER and Golgi^33, 34, 35^ and also in the maintenance of ER morphology.^36^ Previously, we revealed that Yip1A regulates activation of the IRE1 pathway of the UPR and subsequent UPR-induced autophagy under ER stress conditions.^32^

In the present study, we explored the possible role of Yip1A in activation of the UPR by cancer cells. We demonstrated that Yip1A was involved in the constitutive activation of IRE1 and PERK signaling of the UPR in HeLa and CaSki cervical cancer cells, thereby upregulating the expression of anti-apoptotic proteins and autophagy-related proteins. Depletion of Yip1A induced apoptotic cell death in these cancer cells. We propose how IRE1 and PERK signaling pathways contribute coordinately to the survival of HeLa and CaSki cells, and present Yip1A as a key regulatory molecule for the survival of cervical cancer cells.

## Results

### Depletion of Yip1A induced apoptotic cell death in HeLa and CaSki cervical cancer cells

Given that Yip1A is responsible for IRE1 activation,^32^ we hypothesized that Yip1A depletion inhibits IRE1 signaling of the UPR, resulting in unresolved ER stress and apoptosis in cancer cells. To explore this possibility, we knocked down Yip1A expression in HeLa and CaSki cervical cancer cells by using small interfering RNA (siRNA). Depletion of Yip1A was validated by RT-PCR and western blotting (Supplementary Figures 1A and B). The knockdown efficiency of Yip1A protein at 24 h and 48 h after Yip1A-siRNA transfection in HeLa cells was 86±5% and 92±5%, respectively (Supplementary Figure 1B). In CaSki cells, no significant decrease in the amounts of Yip1A protein was observed at 24 h, but the expression of Yip1A protein was decreased by 52±10% and 82±5% at 48 h and 72 h, respectively, after transfection with Yip1A siRNA.

**Figure 1:**
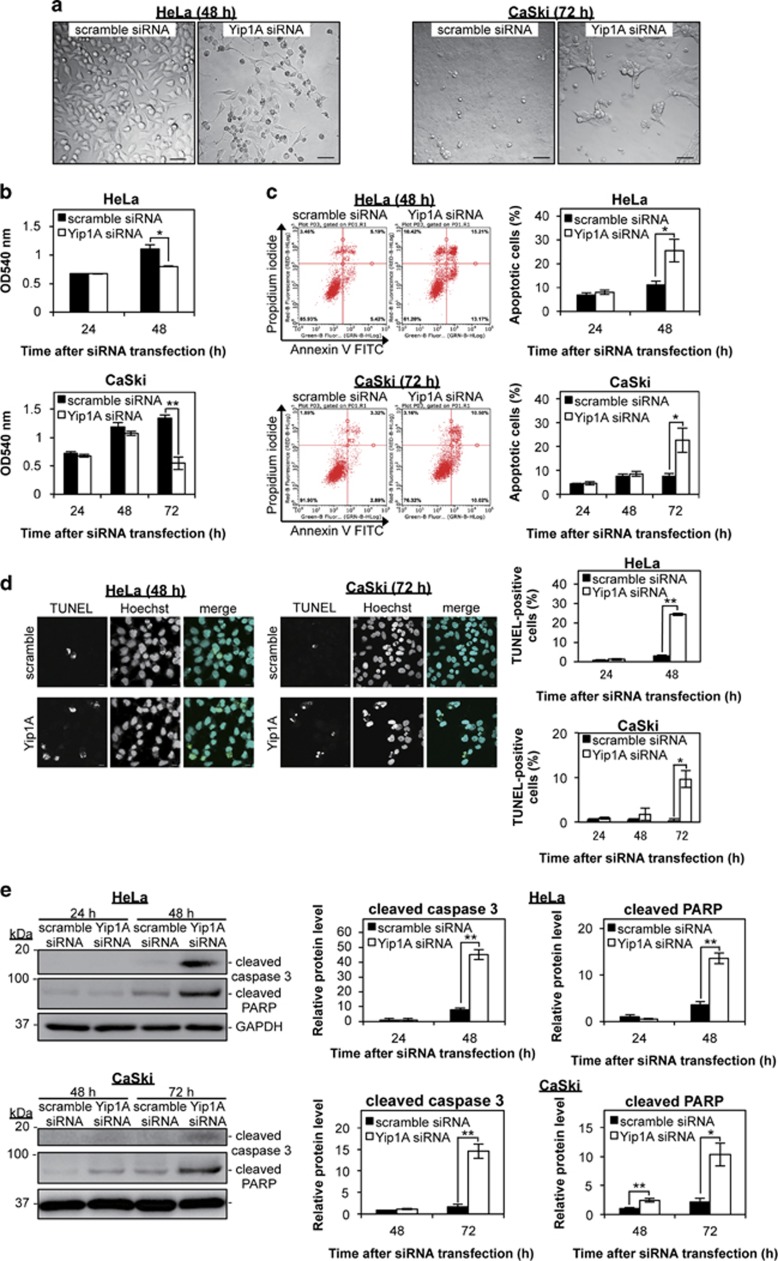
Depletion of Yip1A induces apoptotic cell death in HeLa and CaSki cervical cancer cells. (**a**) HeLa and CaSki cells were transfected with control scramble siRNA (left panel) or Yip1A siRNA (right panel). Representative confocal micrographs show morphological differences between control and Yip1A-knockdown cells at the indicated time points. Scale bars are 50 *μ*m. (**b**) The viability of transfected cells was evaluated at the indicated time points using the MTT Cell Proliferation Assay. Data are means±S.D. from three independent experiments; *P<0.05, **P<0.01. (**c**) Representative flow cytometric data for control (left panels) and Yip1A-knockdown (right panels) cells at the indicated time points after siRNA transfection. The percentages of apoptotic cells (Annexin V^+^/PI^−^ + Annexin V^+^/PI^+^) are shown in the bar graphs. Data are means±S.D. from three independent experiments; *P<0.05. (**d**) Representative confocal micrographs of control (upper panels) and Yip1A-knockdown (lower panels) cells at the indicated time points after siRNA transfection. Scale bars are 10 *μ*m. The percentages of TUNEL-positive cells are shown in the bar graphs. Data are means±S.D. from three independent experiments; *P<0.05, **P<0.01. (**e**) Western blotting shows relative levels of cleaved caspase 3 and cleaved PARP protein in control and Yip1A-knockdown cells at the indicated time points after siRNA transfection. GAPDH was used for normalization. Data are means±S.D. from three independent experiments; *P<0.05, **P<0.01

First, we examined how Yip1A knockdown affected cellular morphology. As seen in Figure 1a, Yip1A-knockdown cells showed apparent cell death at 48 h after siRNA transfection in HeLa cells and at 72 h in CaSki cells. Depletion of Yip1A caused drastic morphological changes and a marked decrease in cell number. The viability of transfected cells was evaluated using the MTT Cell Proliferation Assay (Figure 1b). Compared with control cells, Yip1A-knockdown cells displayed a significant loss of viable cells at 48 h in HeLa cells and at 72 h in CaSki cells. Next, we investigated whether Yip1A knockdown triggered apoptotic cell death by using Annexin V and TUNEL assays. Both flow cytometric analysis (Figure 1c) and TUNEL staining (Figure 1d) revealed that Yip1A depletion increased the total percentages of apoptotic cells at 48 h in HeLa cells and at 72 h in CaSki cells. Apoptotic cell death was not observed in control or Yip1A-knockdown cells at 24 h in HeLa cells and at 48 h in CaSki cells (Supplementary Figures 2A–C). Induction of apoptosis by Yip1A knockdown was further assessed by analyzing caspase 3 and poly (ADP-ribose) polymerase (PARP) processing. Under ER stress-induced apoptosis, caspase 3 is cleaved to its active form, which then cleaves PARP.^37^ Marked increases in the levels of cleaved caspase 3 and PARP products were observed at 48 h after the transfection of Yip1A siRNA in HeLa cells and at 72 h in CaSki cells (Figure 1e). Taken together, these results clearly indicated that Yip1A depletion led to ER stress-induced apoptotic cell death in HeLa and CaSki cervical cancer cells.

### Yip1A knockdown inhibited IRE1 and PERK activation

**Figure 2:**
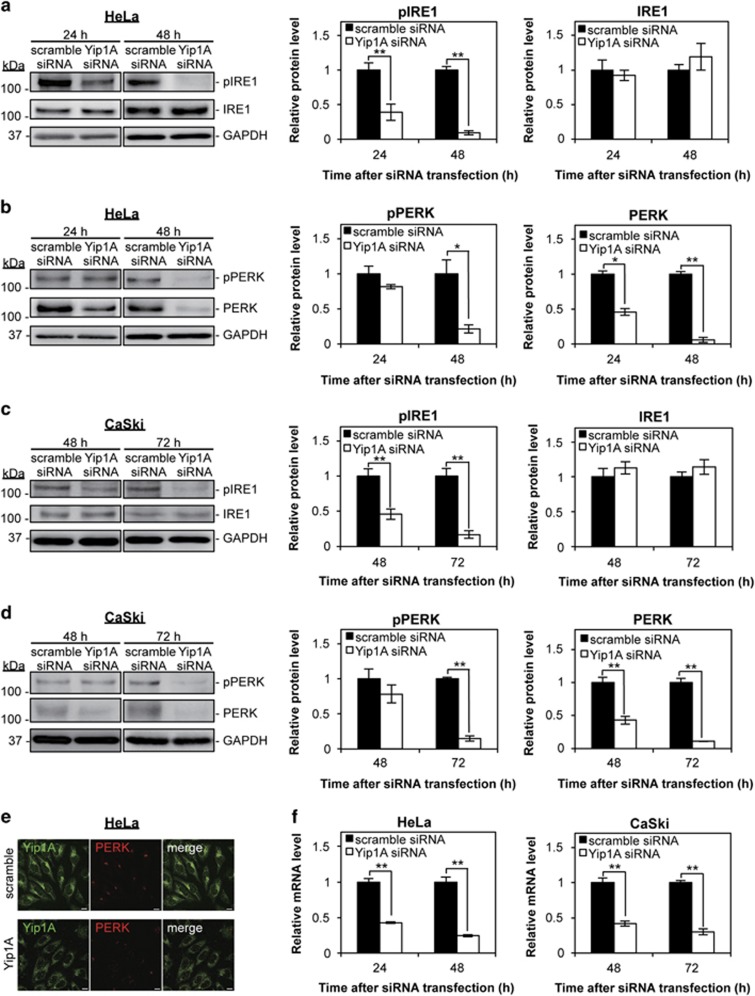
Depletion of Yip1A inhibits the activation of IRE1 and PERK. (**a**) HeLa cells were treated with control scramble siRNA or Yip1A siRNA and cell lysates were prepared at the indicated time points. Western blotting shows the relative levels of phosphorylated IRE1 (pIRE1) and IRE1 protein in control and Yip1A-knockdown cells at 24 h and 48 h after siRNA transfection. GAPDH was used for normalization. Data are means±S.D. from three independent experiments; **P<0.01. (**b**) Western blotting shows relative levels of phosphorylated PERK (pPERK) and PERK protein in control and Yip1A-knockdown cells at 24 h and 48 h after siRNA transfection. GAPDH was used for normalization. Data are means±S.D. from three independent experiments; *P<0.05, **P<0.01. (**c**) CaSki cells were treated with control scramble siRNA or Yip1A siRNA and the cell lysates were prepared at the indicated time points. Western blotting shows the relative levels of pIRE1 and IRE1 protein in control and Yip1A-knockdown cells at 48 h and 72 h after siRNA transfection. GAPDH was used for normalization. Data are means±S.D. from three independent experiments; **P<0.01. (**d**) Western blotting shows relative levels of pPERK and PERK protein in control and Yip1A-knockdown cells at 48 h and 72 h after siRNA transfection. GAPDH was used for normalization. Data are means±S.D. from three independent experiments; **P<0.01. (**e**) Representative confocal micrographs of control (upper panels) and Yip1A-knockdown (lower panels) HeLa cells at 24 h after siRNA transfection, showing the depletion of PERK by Yip1A knockdown. Fixed cells were double-stained for Yip1A (green) and PERK (red). Scale bars are 10 *μ*m. (**f**) RT-PCR shows relative levels of PERK mRNA in control and Yip1A-knockdown cells. GAPDH was used for normalization. Data are means±S.D. from three independent experiments. **P<0.01

Next, we investigated the effects of Yip1A knockdown on the activation of IRE1 and PERK. The levels of phosphorylated IRE1 (pIRE1) were decreased significantly at 24 h, and further lowered at 48 h in Yip1A-knockdown HeLa cells (Figure 2a). There was no significant difference in the total IRE1 levels between control and Yip1A-knockdown cells throughout the experiment (Figure 2a). Notably, a marked decrease in the levels of phosphorylated PERK (pPERK) was observed at 48 h after Yip1A-siRNA transfection in HeLa cells (Figure 2b). In addition, total PERK protein was already reduced at 24 h and had almost disappeared at 48 h after Yip1A knockdown. Similar results were obtained at 48 h and 72 h after siRNA transfection in CaSki cells (Figures 2c and d). The depletion of PERK protein was confirmed by immunofluorescence microscopy in HeLa cells (Figure 2e), which showed obvious loss of PERK protein in Yip1A-knockdown cells. RT-PCR showed a pronounced decrease in PERK mRNA in Yip1A-knockdown cells (Figure 2f), suggesting that Yip1A depletion suppressed PERK transcription. Collectively, these results demonstrated that Yip1A knockdown inhibited IRE1 phosphorylation and attenuated PERK transcription, which would turn off signaling by IRE1, and then PERK. These data also provided evidence that Yip1A was responsible for the constitutive activation of IRE1 and PERK in HeLa and CaSki cells.

### Yip1A knockdown impaired signaling downstream of the IRE1 and PERK pathways

**Figure 3:**
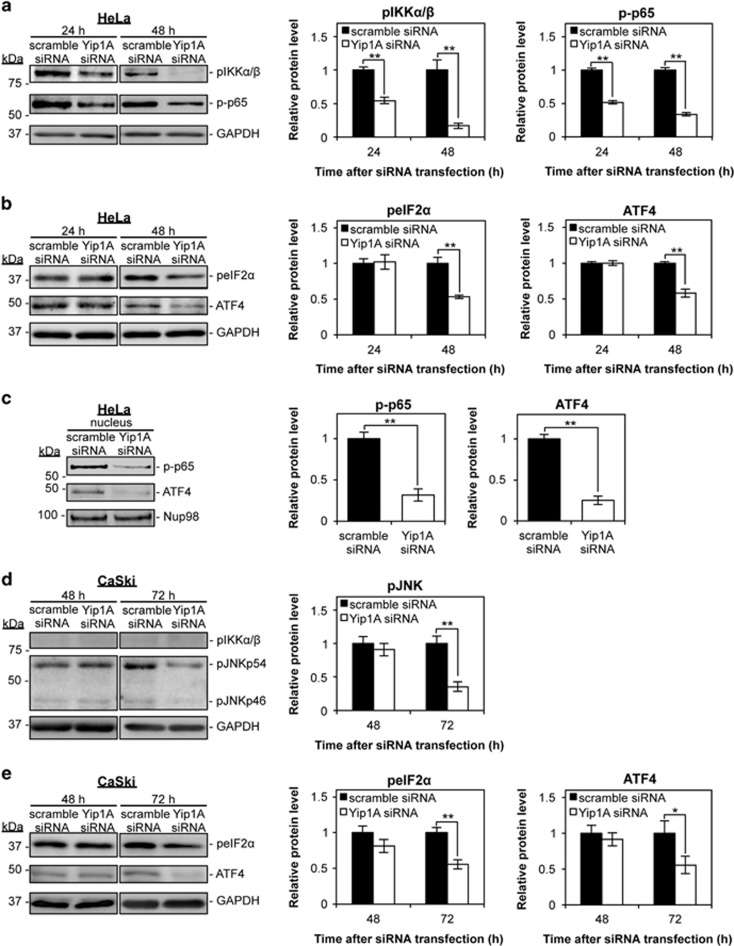
Depletion of Yip1A impairs signaling downstream of the IRE1 and PERK pathways. (**a**) HeLa cells were treated with control scramble siRNA or Yip1A siRNA and the cell lysates were prepared at the indicated time points. Western blotting shows the relative levels of phosphorylated IKK*α/β* (pIKK*α/β*) and phosphorylated NF-*κ*B p65 (p-p65) protein in control and Yip1A-knockdown cells at 24 h and 48 h after siRNA transfection. GAPDH was used for normalization. Data are means±S.D. from three independent experiments; **P<0.01. (**b**) Western blotting shows the relative levels of phosphorylated eIF2*α* (peIF2*α*) and ATF4 protein in control and Yip1A-knockdown cells at 24 h and 48 h after siRNA transfection. GAPDH was used for normalization. Data are means±S.D. from three independent experiments; **P<0.01. (**c**) Western blotting shows relative levels of p-p65 and ATF4 protein in the nuclear fraction of control and Yip1A-knockdown HeLa cells at 48 h after siRNA transfection. Nup98 was used as a control for loading of the nuclear fraction. Data are means±S.D. from three independent experiments; **P<0.01. (**d**) CaSki cells were treated with control scramble siRNA or Yip1A siRNA and the cell lysates were prepared at the indicated time points. Western blotting shows the relative levels of pIKK*α/β* and phosphorylated JNK (pJNKp46 and pJNKp54) protein in control and Yip1A-knockdown cells at 48 h and 72 h after siRNA transfection. GAPDH was used for normalization. Data are means±S.D. from three independent experiments; **P<0.01. (**e**) Western blotting shows the relative levels of peIF2*α* and ATF4 protein in control and Yip1A-knockdown cells at 48 h and 72 h after siRNA transfection. GAPDH was used for normalization. Data are means±S.D. from three independent experiments. *P<0.05, **P<0.01

To characterize the effects of Yip1A knockdown on the IRE1 and PERK pathways further, we investigated signaling downstream of these pathways by western blotting. As can be seen in Figure 3a, Yip1A knockdown blocked IRE1–IKK–NF-*κ*B signaling in HeLa cells. The levels of phosphorylated IKK*α/β* (pIKK*α/β*) and phosphorylated NF-*κ*B p65 (p-p65) were decreased markedly at 24 h, and further lowered at 48 h after Yip1A-siRNA transfection (Figure 3a). Attenuation of PERK signaling was also evident in Yip1A-knockdown HeLa cells at 48 h (Figure 3b). Phosphorylation of eIF2*α* (peIF2*α*) and translation of ATF4 were reduced at 48 h after Yip1A knockdown. The amounts of total IKK*α/β*, p65, and eIF2*α* protein did not differ significantly between control and Yip1A-knockdown cells throughout these experiments (Supplementary Figure 3A). Then, nuclear fractions from siRNA-transfected HeLa cells were prepared at 48 h after transfection, and the levels of the transcription factors p-p65 and ATF4 in the nucleus examined. Although substantial levels of these prosurvival transcription factors were seen in the nuclei of control cells, they were virtually undetectable in the nuclear fractions of Yip1A-knockdown cells (Figure 3c).

In contrast, pIKK*α/β* was barely observed at 48 h and 72 h after siRNA transfection in CaSki cells, and no detectable difference was found between the control and Yip1A-knockdown cells (Figure 3d). Instead, a marked decrease in the levels of phosphorylated JNK proteins (pJNKp46 and pJNKp54) was observed at 72 h after Yip1A-siRNA transfection (Figure 3d), indicating that IRE1–JNK signaling may be interrupted by the depletion of Yip1A in CaSki cells. There was no significant difference in the amount of pJNKp46 and pJNKp54 proteins in HeLa cells (Supplementary Figure 3A). PERK–peIF2*α*–ATF4 signaling was also suppressed at 72 h in CaSki cells (Figure 3e). No significant difference in the amounts of total eIF2*α* protein was detected between the control and Yip1A-knockdown cells (Supplementary Figure 3B). Thus, Yip1A knockdown impaired signaling downstream of the IRE1 and PERK pathways in HeLa and CaSki cells.

### Yip1A knockdown downregulated anti-apoptotic proteins

**Figure 4:**
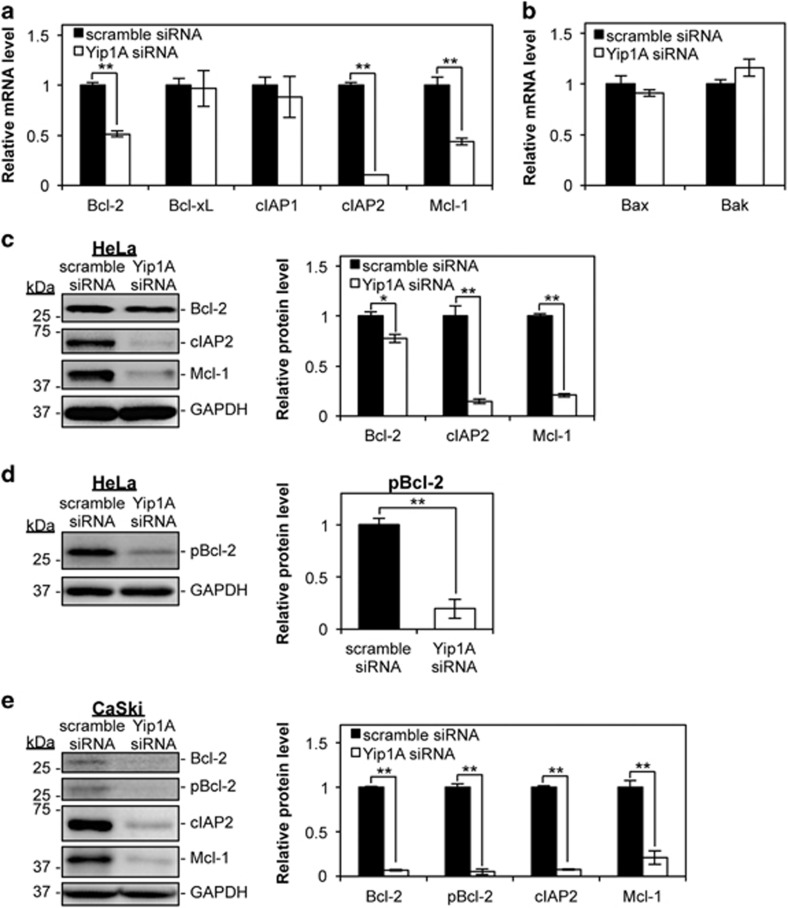
Depletion of Yip1A downregulates the expression of the anti-apoptotic proteins Bcl-2, cIAP2, and Mcl-1, and the phosphorylation of Bcl-2. (**a**) HeLa cells were transfected with control scramble siRNA or Yip1A siRNA. RT-PCR shows the relative mRNA levels of the indicated anti-apoptotic factors in control and Yip1A-knockdown cells at 48  after siRNA transfection. GAPDH was used for normalization. Data are means±S.D. from three independent experiments; **P<0.01. (**b**) RT-PCR shows the relative mRNA levels of the indicated proapoptotic factors in control and Yip1A-knockdown HeLa cells at 48 h after siRNA transfection. GAPDH was used for normalization. Data are means±S.D. from three independent experiments. (**c**) Western blotting shows the relative levels of anti-apoptotic Bcl-2, cIAP2, and Mcl-1 protein in control and Yip1A-knockdown cells at 48 h after siRNA transfection. GAPDH was used for normalization. Data are means±S.D. from three independent experiments; *P<0.05, **P<0.01. (**d**) Western blotting shows the relative levels of phosphorylated Bcl-2 (pBcl-2) protein in control and Yip1A-knockdown cells at 48 h after siRNA transfection. GAPDH was used for normalization. Data are means±S.D. from three independent experiments; **P<0.01. (**e**) CaSki cells were transfected with control scramble siRNA or Yip1A siRNA. Western blotting shows the relative levels of anti-apoptotic Bcl-2, pBcl-2, cIAP2, and Mcl-1 protein in control and Yip1A-knockdown cells at 72 h after siRNA transfection. GAPDH was used for normalization. Data are means±S.D. from three independent experiments; **P<0.01

Downregulation of anti-apoptotic proteins and/or upregulation of proapoptotic proteins are likely to tilt the cellular balance from survival to apoptosis under ER stress. We investigated the expression of these proteins during Yip1A knockdown in HeLa cells. At 48 h after siRNA transfection, RT-PCR revealed a marked reduction in the mRNA levels of anti-apoptotic Bcl-2, cellular inhibitor of apoptosis protein 2 (cIAP2), and myeloid cell leukemia-1 (Mcl-1) in Yip1A-knockdown cells relative to control cells (Figure 4a). No significant differences in the mRNA levels of two major proapoptotic Bax and Bak were observed (Figure 4b). The downregulation of the anti-apoptotic proteins was further confirmed by western blotting (Figure 4c). Then, we examined the phosphorylation of Bcl-2 at Serine 70 (Ser70), which is required for its full activation.^38^ The amount of phosphorylated Bcl-2 (pBcl-2) was decreased drastically by Yip1A knockdown (Figure 4d). In CaSki cells, a pronounced reduction in the levels of these anti-apoptotic proteins was observed at 72 h after Yip1A-siRNA transfection (Figure 4e). Altogether, these results indicated that downregulation of the anti-apoptotic proteins Bcl-2, cIAP2, and Mcl-1, as well as disrupted Bcl-2 phosphorylation, might be responsible for apoptotic cell death induced by Yip1A knockdown.

### Yip1A knockdown suppressed autophagy

**Figure 5:**
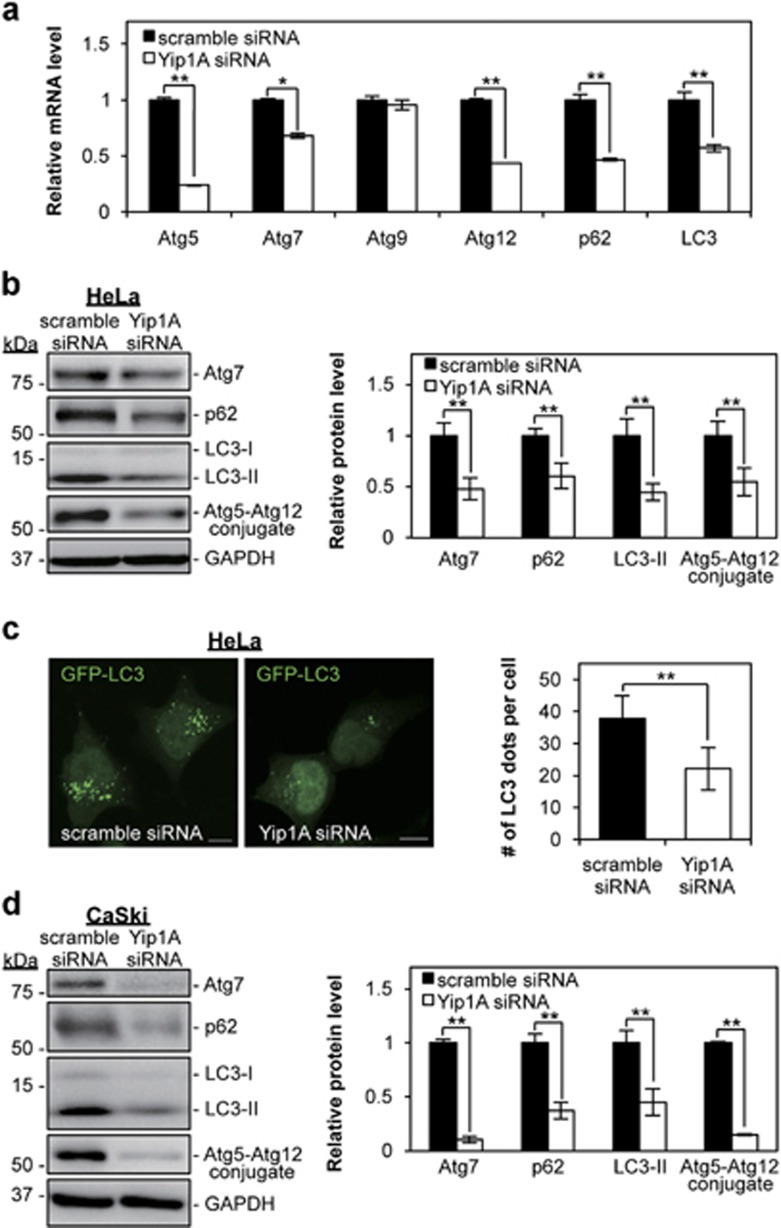
Depletion of Yip1A suppresses autophagy. (**a**) HeLa cells were transfected with control scramble siRNA or Yip1A siRNA. RT-PCR shows the relative mRNA levels of the indicated autophagy-related factors in the control and Yip1A-knockdown cells at 48 h after siRNA transfection. GAPDH was used for normalization. Data are means±S.D. from three independent experiments; *P<0.05, **P<0.01. (**b**) Western blotting shows the relative levels of Atg7, p62, and LC3-II protein, and Atg5–Atg12 conjugate in the control and Yip1A-knockdown cells at 48 h after siRNA transfection. GAPDH was used for normalization. Data are means±S.D. from three independent experiments; **P<0.01. (**c**) HeLa cells were co-transfected with either control scramble siRNA or Yip1A siRNA and pEGFP-LC3 expression plasmid DNA. After 48 h, autophagosome formation was measured by visualizing LC3-positive dots with the use of a confocal microscope. Scale bars are 10 *μ*m. Quantification of the number of LC3-positive dots per cell is shown in the bar graph (*n*=30 cells); **P<0.01. (**d**) CaSki cells were transfected with control scramble siRNA or Yip1A siRNA. Western blotting shows the relative levels of Atg7, p62, and LC3-II protein, and Atg5–Atg12 conjugate in the control and Yip1A-knockdown cells at 72 h after siRNA transfection. GAPDH was used for normalization. Data are means±S.D. from three independent experiments; **P<0.01

UPR-induced autophagy is one mechanism that can alleviate ER stress.^20, 39, 40, 41^ Recent studies have implicated the IRE1–JNK pathway and the PERK–eIF2*α*–ATF4 pathway in the induction of autophagy.^20, 23^ First, we investigated the effects of Yip1A depletion on the expression of autophagy-related proteins in HeLa cells. RT-PCR was performed for selected autophagy-related genes at 48 h after siRNA transfection. Yip1A knockdown resulted in downregulation of Atg5, Atg7, Atg12, p62, and LC3 (Figure 5a). The drastic decrease in Atg7, p62, and LC3 expression was confirmed at the protein level (Figure 5b). In addition, western blotting revealed that formation of the Atg5–Atg12 conjugate, which indicates the onset of autophagy, was suppressed significantly in the Yip1A-knockdown cells (Figure 5b). Autophagosome formation was further evaluated by fluorescence microscopy. Co-transfection of an expression construct for GFP-LC3 with Yip1A siRNA into HeLa cells enabled LC3-positive vesicles (autophagosomes) to be visualized. At 48 h after co-transfection, considerably lower numbers of LC3-positive dots were observed in the Yip1A-knockdown cells than in the control cells, in which a large number of GFP-LC3 dots could be seen (Figure 5c). In CaSki cells, a marked decrease in the levels of these autophagy-related proteins was detected at 72 h after Yip1A-siRNA transfection (Figure 5d). These results demonstrated that Yip1A depletion led to downregulation of autophagy-related proteins and interfered with autophagosome formation. Also, these results indicated that HeLa and CaSki cells maintained basal levels of autophagy.

### Double depletion of IRE1 and PERK downregulated anti-apoptotic proteins and autophagy-related proteins

**Figure 6:**
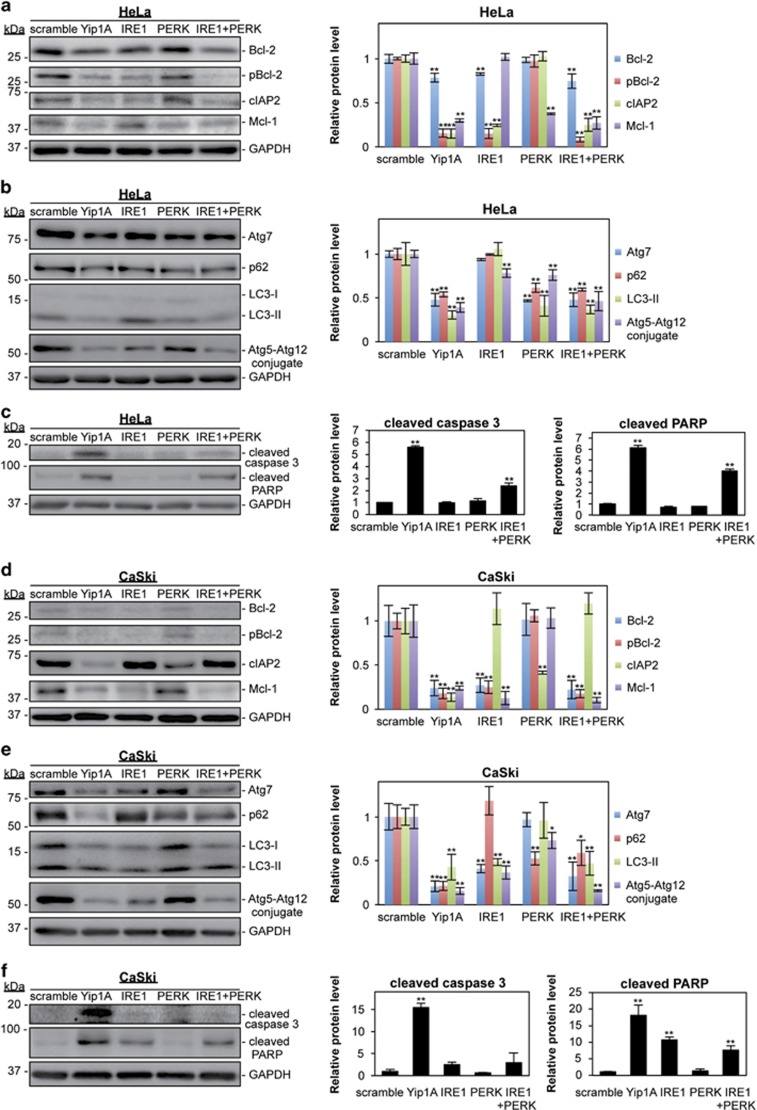
Both the IRE1 and PERK pathways engage in the upregulation of anti-apoptotic proteins and autophagy-related proteins in HeLa and CaSki cells. (**a**) HeLa cells were transfected with the indicated siRNAs, and cell lysates were prepared at 48 h. Western blotting shows the relative levels of Bcl-2, pBcl-2, cIAP2, and Mcl-1 protein in respective siRNA-transfected cells. GAPDH was used for normalization. Data are means±S.D. from three independent experiments. The significant difference from the control (scramble) was determined using Dunnett's *post hoc* test; **P<0.01. (**b**) Western blotting shows relative levels of Atg7, p62, and LC3-II protein, and Atg5–Atg12 conjugate in respective siRNA-transfected HeLa cells. GAPDH was used for normalization. Data are means±S.D. from three independent experiments. The significant difference from the control (scramble) was determined using Dunnett's *post hoc* test; **P<0.01. (**c**) Western blotting shows relative levels of cleaved caspase 3 and cleaved PARP protein in respective siRNA-transfected HeLa cells. GAPDH was used for normalization. Data are means±S.D. from three independent experiments. The significant difference from the control (scramble) was determined using Dunnett's *post hoc* test; **P<0.01. (**d**) CaSki cells were transfected with the indicated siRNAs, and the cell lysates were prepared at 72 h. Western blotting shows the relative levels of Bcl-2, pBcl-2, cIAP2, and Mcl-1 protein in respective siRNA-transfected cells. GAPDH was used for normalization. Data are means±S.D. from three independent experiments. The significant difference from the control (scramble) was determined using Dunnett's *post hoc* test; **P<0.01. (**e**) Western blotting shows relative levels of Atg7, p62, and LC3-II protein, and Atg5–Atg12 conjugate in respective siRNA-transfected CaSki cells. GAPDH was used for normalization. Data are means±S.D. from three independent experiments. The significant difference from the control (scramble) was determined using Dunnett's *post hoc* test; *P<0.05, **P<0.01. (**f**) Western blotting shows relative levels of cleaved caspase 3 and cleaved PARP protein in respective siRNA-transfected CaSki cells. GAPDH was used for normalization. Data are means±S.D. from three independent experiments. The significant difference from the control (scramble) was determined using Dunnett's *post hoc* test; **P<0.01

To demonstrate that disruption of IRE1 and/or PERK signaling was responsible for the downregulation of anti-apoptotic proteins and autophagy-related proteins induced by Yip1A knockdown, IRE1 and PERK were depleted independently or together using RNA interference (RNAi) in HeLa and CaSki cells. Knockdown of each protein was confirmed by western blotting (Supplementary Figures 4A and B). In HeLa cells, double depletion of IRE1 and PERK (IRE1+PERK) showed similar results to Yip1A knockdown at 48 h after siRNA transfection: downregulated expression of the anti-apoptotic proteins Bcl-2, cIAP2, and Mcl-1 and the autophagy-related proteins Atg7, p62, and LC3, suppressed phosphorylation of Bcl-2, and impaired formation of the Atg5–Atg12 conjugate (Figures 6a and b). Silencing of IRE1 alone was followed by downregulation of Bcl-2 and cIAP2 and by dephosphorylation of Bcl-2, whereas depletion of PERK was associated with downregulation of Mcl-1, Atg7, p62, and LC3 (Figures 6a and b). Intriguingly, the Atg5–Atg12 conjugate decreased partially when either IRE1 or PERK alone were knocked down, but double depletion resulted in levels similar to those seen after Yip1A knockdown (Figure 6b). Then, induction of apoptosis was evaluated using caspase 3 and PARP processing (Figure 6c). Neither IRE1 nor PERK knockdown led to a significant increase in the cleaved products, whereas double knockdown of IRE1 and PERK allowed the generation of these products.

In CaSki cells, the double knockdown of IRE1 and PERK also downregulated anti-apoptotic proteins and autophagy-related proteins, except for cIAP2, at 72 h after Yip1A-siRNA transfection (Figures 6d and e). However, unlike in HeLa cells, IRE1 knockdown reduced the expression of Bcl-2, Mcl-1, Atg7, and LC3, and PERK knockdown decreased the cIAP2 and p62 expression in CaSki cells. Again, only the double depletion of IRE1 and PERK suppressed formation of the Atg5–Atg12 conjugate to levels similar to those after Yip1A knockdown (Figure 6e). Cleaved caspase 3 was generated only by Yip1A-knockdown, and cleaved PARP was detected by IRE1 knockdown and double knockdown (IRE1+PERK) as well as by Yip1A-knockdown (Figure 6f). These results indicated that the IRE1 and PERK pathways coordinately engage in the upregulation of anti-apoptotic proteins and autophagy-related proteins, and that Yip1A might function as a key modulator to activate both pathways for survival.

## Discussion

The UPR comprised several signaling pathways that modulate cell fate between survival and apoptosis on an increase in ER stress. Constitutive activation of the UPR has been reported as a prosurvival strategy for numerous cancer cells.^1, 2, 3, 4^ However, the molecular switches that mediate transition from apoptosis to survival remain to be elucidated. To our knowledge, this study is the first to identify a key prosurvival modulator, Yip1A, which coordinates IRE1 and PERK signaling to support the survival of cervical cancer cells. Possible prosurvival roles of Yip1A are to prevent ER stress-induced apoptosis through promoting anti-apoptotic signals, and provide a survival advantage by enhancing autophagy. Yip1A depletion triggers ER stress-induced apoptotic cell death by disrupting the prosurvival signaling of the UPR and enhancing severe ER stress.

**Figure 7:**
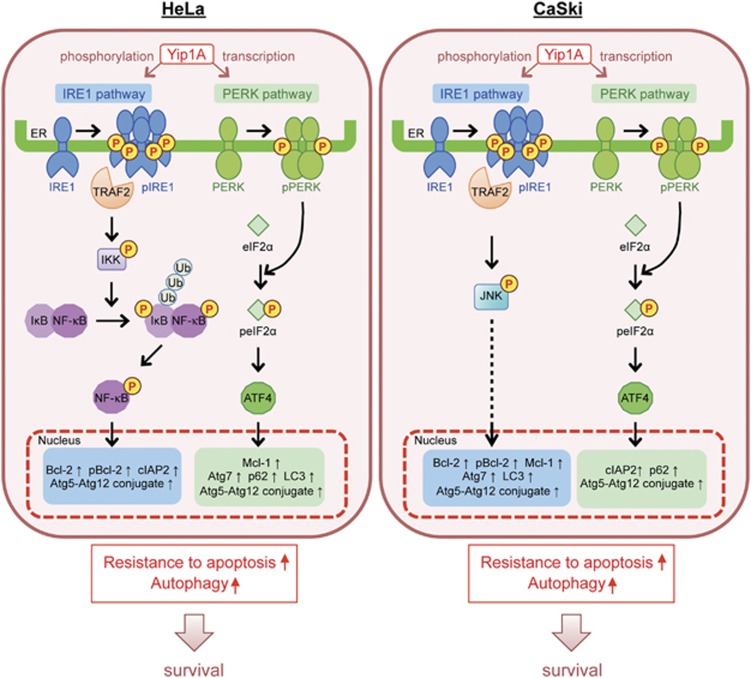
Schematic representation of how Yip1A operates as a prosurvival modulator that coordinately activates the IRE1 and PERK pathways of the UPR to support the survival of HeLa and CaSki cervical cancer cells. See text for details. Ub, ubiquitination

Many studies on the UPR have been performed using pharmacological agents to induce ER stress, such as tunicamycin, thapsigargin, dithiothreitol, and brefeldin A. However, these agents cause additional effects on cells, and caution should be taken in interpreting the results. In this study, we compared cells transfected with Yip1A siRNA with those transfected with control scramble siRNA in the absence of stimuli, as basal constitutive activation of IRE1 and PERK can be detected in HeLa and CaSki cells (Figure 2). From the findings obtained, we have proposed a model for how Yip1A functions as a prosurvival modulator that coordinately activates the IRE1 and PERK pathways of the UPR to support the survival of HeLa and CaSki cervical cancer cells (Figure 7). We showed that Yip1A promoted the constitutive activation of IRE1 and PERK signaling of the UPR in HeLa and CaSki cells (Figure 2). Yip1A mediated IRE1 phosphorylation and also induced PERK transcription. The activation of IRE1 appeared to transduce signals through the IRE1–IKK–NF-*κ*B pathway in HeLa cells, and through the IRE1–JNK pathway in CaSki cells (Figure 3). The PERK–eIF2*α*–ATF4 pathway was activated by Yip1A in HeLa and CaSki cells. Consequently, expression of the anti-apoptotic proteins Bcl-2, cIAP2, and Mcl-1 and phosphorylation of Bcl-2 were upregulated (Figure 4), thereby promoting resistance to apoptosis under ER stress. Increased expression of the autophagy-related proteins Atg5, Atg7, Atg12, p62, and LC3 enhanced autophagy (Figure 5), which would degrade inadequate protein aggregates and also provide nutrients for rapidly growing cancer cells. Thus, Yip1A operates as a prosurvival modulator that coordinately activates the IRE1 and PERK pathways of the UPR to support the survival of HeLa and CaSki cervical cancer cells.

Recent studies have suggested that the regulation of cell fate by the UPR is so complex that it might require cross talk between several signaling pathways.^12, 42^ The IRE1 and PERK pathways appear to have complementary functions, and silencing of either pathway is insufficient to cause ER stress-induced apoptosis. For example, the UPR degrades and removes unfolded protein aggregates by ERAD and autophagy. Constitutively activated IRE1 signaling upregulates target genes involved in ERAD. Inhibiting IRE1 signaling impairs this degradation system, but cells can compensate for decreased ERAD by activating autophagy through PERK signaling to alleviate ER stress.^43^ Therefore, it might be necessary to disrupt these pathways simultaneously. Yip1A knockdown is unique in that it disrupts both IRE1 and PERK signaling but in different ways. Yip1A depletion represses IRE1 phosphorylation, and this is accompanied by suppression of PERK transcription. We demonstrated that the shutoff of IRE1 signaling preceded the interruption of PERK signaling (Figure 2). This sequential timing should be advantageous because it prevents complementation between the IRE1 and PERK pathways. The ability of Yip1A knockdown to block IRE1 and PERK signaling successively allows global protein synthesis to continue in the face of impaired ERAD and autophagy, thus increasing the accumulation of unresolved protein aggregates in the ER. In addition, the marked reduction in anti-apoptotic proteins should tilt the cellular balance from survival to apoptosis. Thus, Yip1A knockdown drives HeLa and CaSki cells toward ER stress-induced apoptosis.

We found that HeLa and CaSki cells maintained basal levels of autophagy (Figure 5). There is increasing evidence that UPR-induced autophagy is crucial for cancer cell survival.^44^ However, the UPR signaling pathways that support autophagy under ER stress remain to be established. Some studies demonstrate a requirement for PERK signaling,^45, 46, 47^ whereas others indicate that IRE1 signaling is required for UPR-mediated autophagy.^20, 48, 49^ Notably, our study revealed that IRE1 and PERK signaling functioned coordinately in inducing autophagy. The results showed that formation of the Atg5–Atg12 conjugate required both IRE1 and PERK signaling. With respect to IRE1 signaling, we speculate that IRE1 might be required for the onset of autophagy. We demonstrated previously that IRE1 molecules form higher-order assemblies with Yip1A under ER stress, which supports the formation of ER-derived autophagic vacuoles.^32^ Several recent studies suggest that IRE1 oligomers formed on ER stress operate as a molecular platform to interact with regulators that control downstream signaling.^42^ Yip1A might function as such a regulator to mediate cross talk between the UPR and autophagy. The present study also sheds light on cancers showing resistance to therapies. The UPR and autophagy are known to be activated following cancer therapies, such as chemotherapy and radiotherapy, and protect cancer cells from ER stress-induced apoptotic cell death.^50, 51, 52, 53^ The marked suppression of IRE1 and PERK signaling of the UPR and UPR-induced autophagy by Yip1A knockdown provides evidence that targeting Yip1A has potential to overcome apoptosis resistance and to enhance the sensitivity of cancer cells to anticancer treatments.

In conclusion, this study uncovered a novel modulatory role of Yip1A in IRE1 and PERK signaling of the UPR for the survival of HeLa and CaSki cells. Elucidation of Yip1A functions in cancer cells will provide insights into the molecular mechanisms by which cancer cells avoid ER stress-induced apoptosis.

## Materials and methods

### Cell culture

HeLa cells (previously existing collection in the Murata Laboratory at the University of Tokyo) were cultured in Dulbecco's Modified Eagle's Medium (DMEM; Nissui, Tokyo, Japan) supplemented with 10% fetal bovine serum (FBS; Sigma-Aldrich, St. Louis, MO, USA) and penicillin/streptomycin (Gibco, Thermo Fisher Scientific, Waltham, MA, USA) under conditions of 5% CO_2_ atmosphere at 37 °C. A human cervical cancer cell line CaSki (IFO50007)^54^ was obtained from the Japanese Collection of Research Bioresources (JCRB, Osaka, Japan), and grown in RPMI 1640 Medium (Nissui) supplemented with 10% FBS (Sigma-Aldrich) and 2 mM l-glutamine (Gibco) under conditions of 5% CO_2_ atmosphere at 37 °C. For transfection, HeLa cells were seeded into DMEM supplemented with 10% FBS, and CaSki cells were seeded into RPMI 1640 Medium supplemented with 10% FBS and 2 mM l-glutamine in 35-mm culture dishes. For confocal microscopy, the cells were plated onto coverslips in 35 mm culture dishes. Cell morphology was examined on a Zeiss LSM 510 laser scanning confocal microscope (Carl Zeiss, Oberkochen, Germany) with a × 20 objective lens.

### Antibodies, plasmid, and siRNAs

The primary antibodies used were: Caspase-3 (8G10) Rabbit mAb (#9665), PARP antibody (#9542), Phospho-IKK*α* (Ser176)/IKK*β* (Ser177) (C84E11) Rabbit mAb (#2078), IKK*α* antibody (#2682), IKK*β* (2C8) Rabbit mAb (#2370), Phospho-NF-*κ*B p65 (Ser536) (93H1) Rabbit mAb (#3033), NF-*κ*B p65 (D14E12) XP Rabbit mAb (#8242), Phospho-eIF2*α* (Ser51) antibody (#9721), eIF2*α* antibody (#9722), ATF4 (D4B8) Rabbit mAb (#11815), Phospho-SAPK/JNK (Thr183/Tyr185) antibody (#9251), Bcl-2 (50E3) Rabbit mAb (#2870), Phospho-Bcl-2 (Ser70) (5H2) Rabbit mAb (#2827), cIAP2 (58C7) Rabbit mAb (#3130), Mcl-1 (D35A5) Rabbit mAb (#5453), Atg7 antibody (#2631), and Atg12 (D88H11) Rabbit mAb (#4180) (all from Cell Signaling Technology, Danvers, MA, USA); Anti-glyceraldehyde-3-phosphate dehydrogenase antibody, Clone 6C5 (#MAB374, Merck Millipore Corporation, Darmstadt, Germany); Nup98 (C-5) mouse monoclonal antibody (#sc-74578, Santa Cruz Biotechnology, Santa Cruz, CA, USA); Anti-LC3 pAb (#PM036, MBL, Nagoya, Japan); Anti-p62 (SQSTM1) pAb (#PM045, MBL), Anti-IRE1 (phospho S724) antibody (#ab48187, Abcam, Cambridge, UK); Anti-IRE1 antibody (#ab37073, Abcam); Anti-PERK antibody (#ab65142, Abcam); and pPERK (Thr981) (#sc-32577, Santa Cruz Biotechnology). The rabbit polyclonal anti-Yip1A antibody was raised as described in Kano *et al.*^35^ The guinea pig polyclonal anti-Yip1A antibody was generated as described in Taguchi *et al.*^32^ The secondary antibodies used for immunofluorescence were: Goat anti-Guinea Pig IgG (H+L) Secondary Antibody, Alexa Fluor 488 conjugate (#A-11073, Life Technologies, Thermo Fisher Scientific), and Goat anti-Rabbit IgG (H+L) Secondary Antibody, Alexa Fluor 568 (#A-11011, Life Technologies). The secondary antibodies used for western blotting were: Anti-Mouse IgG (H+L), HRP Conjugate (#4021, Promega, Madison, WI, USA), Goat anti-Guinea pig IgG H&L (HRP) (#ab6908, Abcam), and Anti-Rabbit IgG, HRP-linked Antibody (#7074, Cell Signaling Technology). The pEGFP-LC3 expression plasmid was a gift from Dr. Y Ohsumi (Tokyo Institute of Technology, Yokohama, Japan). Small interfering RNA (siRNA) against Yip1A (#127564) and negative control scramble siRNA (Silencer Negative Control 1 siRNA, #AM4635) were obtained from Ambion (Thermo Fisher Scientific), and siRNAs against IRE1 (SASI_Hs01_00194923) and PERK (SASI_Hs0100096844) were from Sigma-Aldrich.

### Transfections

Lipofectamine RNAiMax Transfection Reagent (Invitrogen, Thermo Fisher Scientific) was used for siRNA transfection in accordance with the manufacturer's protocol. For co-transfection with siRNA and pEGFP-LC3 plasmid DNA, Lipofectamine 2000 Transfection Reagent (Invitrogen) was used in accordance with the manufacturer's protocol.

### MTT assay

Cell viability was evaluated using a CytoSelect MTT Cell Proliferation Assay kit (Cell Biolabs Inc., San Diego, CA, USA) in accordance with the manufacturer's instructions. Absorbance was measured at 540 nm with a microplate reader (Benchmark Plus Microplate Spectrophotometer; Bio-Rad, Hercules, CA, USA).

### Annexin V assay

HeLa and CaSki cells were collected at indicated time points after siRNA transfection, and co-stained with Annexin V-fluorescein isothiocyanate (FITC) and propidium iodide (PI) using an ApoAlert Annexin V-FITC Apoptosis Kit (Clontech, Takara Bio, Shiga, Japan) in accordance with the manufacturer's instructions. Stained cells were analyzed using a Guava easyCyte Flow Cytometer (Merck Millipore Corporation).

### TUNEL assay

For terminal deoxynucleotidyl transferase dUTP nick-end labeling (TUNEL), HeLa and CaSki cells were stained by using an *In Situ* Cell Death Detection Kit, Fluorescein (Roche Diagnostics GmbH, Mannheim, Germany) in accordance with the manufacturer's protocol. Hoechst 33342 (Dojindo, Kumamoto, Japan) was used to visualize the nucleus. Images were obtained under oil immersion on a Zeiss LSM 510 laser scanning confocal microscope (Carl Zeiss) and Nikon A1 laser scanning confocal microscope (Nikon, Tokyo, Japan). TUNEL-positive cells were counted and expressed as the percentage of apoptotic cells relative to counted cells (*n*=500).

### SDS-PAGE and western blotting

HeLa and CaSki cell lysates were prepared as described previously.^32^ The lysates were subjected to SDS-PAGE, and transferred onto PVDF membrane (Merck Millipore Corporation). The membrane was blocked for 1 h at room temperature with Tris-buffered saline (TBS) that contained 0.1% Tween 20 (TBST) and 5% bovine serum albumin (BSA; Equitech-Bio Inc., Kerrville, TX, USA), and then incubated with relevant primary antibodies in blocking buffer overnight at 4 °C. After washing with TBST, the membrane was incubated with species-specific secondary antibodies in blocking buffer for 1 h at room temperature. After washing with TBST, protein bands were detected using Western Lightning Plus-ECL, Enhanced Chemiluminescence Substrate (PerkinElmer, Inc., Waltham, MA, USA) and a LAS-4000 mini imaging system (GE Healthcare, Little Chalfont, UK). The intensity of the bands was quantified using the MultiGauge software (Fujifilm Inc., Tokyo, Japan).

### Immunofluorescence microscopy

HeLa cells were fixed and permeabilized with methanol–acetone (1:1, v/v) for 6.5 min at 4 °C. After washing with phosphate-buffered saline (PBS), the cells were blocked for 30 min in PBS that contained 3% BSA and then incubated with relevant primary antibodies in blocking buffer for 2 h at room temperature. After washing with PBS, the cells were incubated with fluorescent secondary antibodies in blocking buffer for 1 h at room temperature. The coverslips were mounted in SlowFade Gold Antifade Mountant (Life Technologies) and the images were obtained under oil immersion on a Zeiss LSM 510 laser scanning confocal microscope (Carl Zeiss).

### RNA isolation and RT-PCR

Total RNA was purified from siRNA-treated HeLa cells using an RNeasy Mini Kit (Qiagen, Hilden, Germany) and reverse-transcribed into cDNA with the use of a ReverTra Ace qPCR RT Kit (TOYOBO Co. Ltd., Osaka, Japan) in accordance with each manufacturer's protocol. One-step PCR was carried out using Fast SYBR Green Master Mix (Applied Biosystems, Thermo Fisher Scientific) and a StepOnePlus Real-Time PCR System (Applied Biosystems) following the manufacturer's instructions. Primer pairs used for PCR amplification are listed in Supplementary Table 1. Glyceraldehyde-3-phosphate dehydrogenase (GAPDH) was used as an internal standard.

### Cell fractionation

At 48 h after siRNA transfection, nuclear and cytosol fractions were extracted using a Nuclear/Cytosol Fractionation Kit (BioVision Inc., Milpitas, CA, USA) in accordance with the manufacturer's protocol. Equal amounts of protein were loaded for western blotting.

### Statistical analysis

Data are expressed as the mean±standard deviation (S.D.). The statistical significance of differences observed in individual sets of data was assessed using Welch's *t*-test except Figures 6a–f and Supplementary Figures 4A and 4B where the significant difference from the control was determined using Dunnett's *post hoc* test. Differences were considered statistically significant at *P*<0.05.
