# Supplementary figures and images for: Novel prosurvival function of Yip1A in human cervical cancer cells: constitutive activation of the IRE1 and PERK pathways of the unfolded protein response
Source: Cell Death Dis. 2017 Mar 30;8(3):e2718–. doi: 10.1038/cddis.2017.147 (PMC5386543; doi:10.1038/cddis.2017.147)

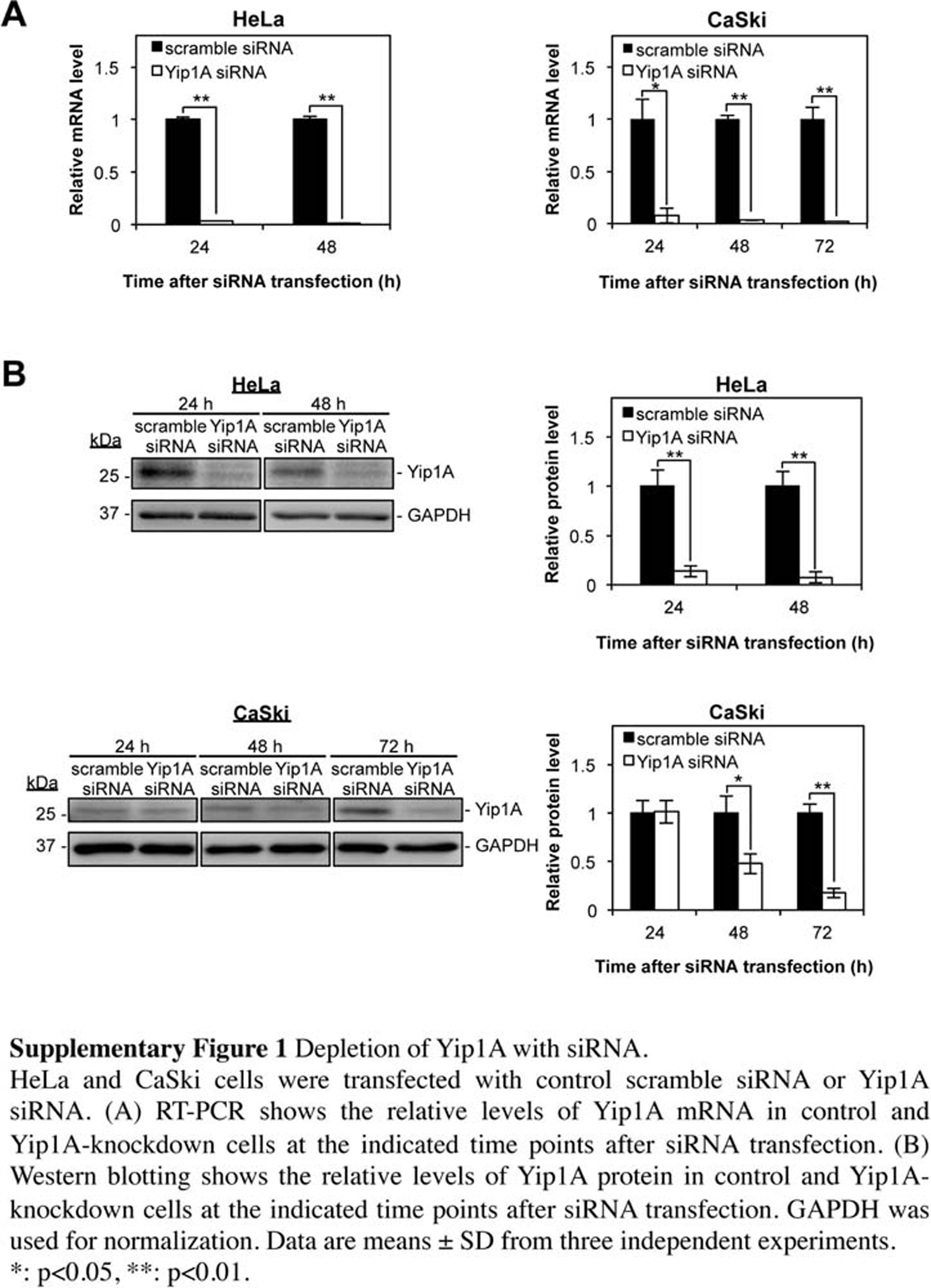

Supplement: Supplementary Figure 1 [file cddis2017147x1.tif]

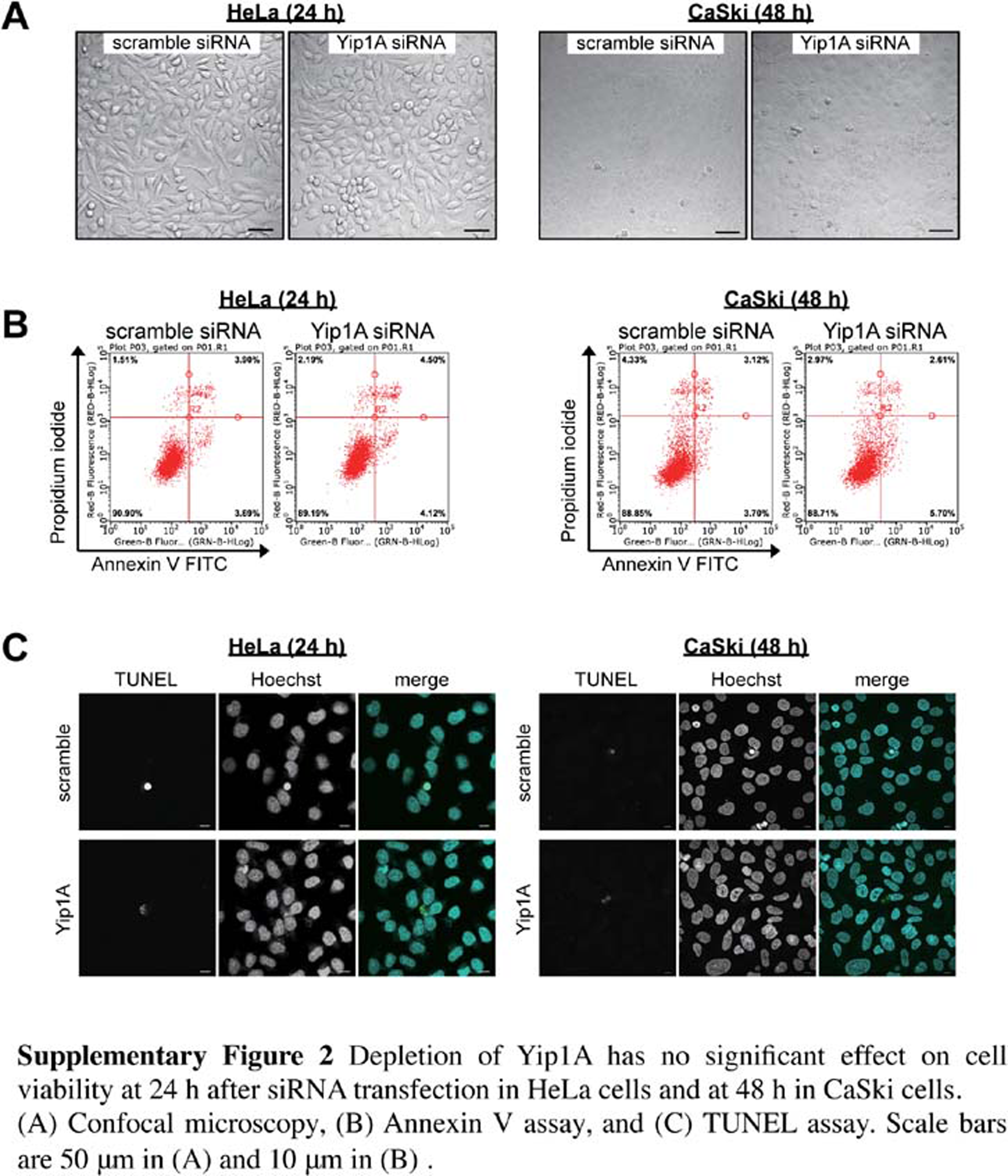

Supplement: Supplementary Figure 2 [file cddis2017147x2.tif]

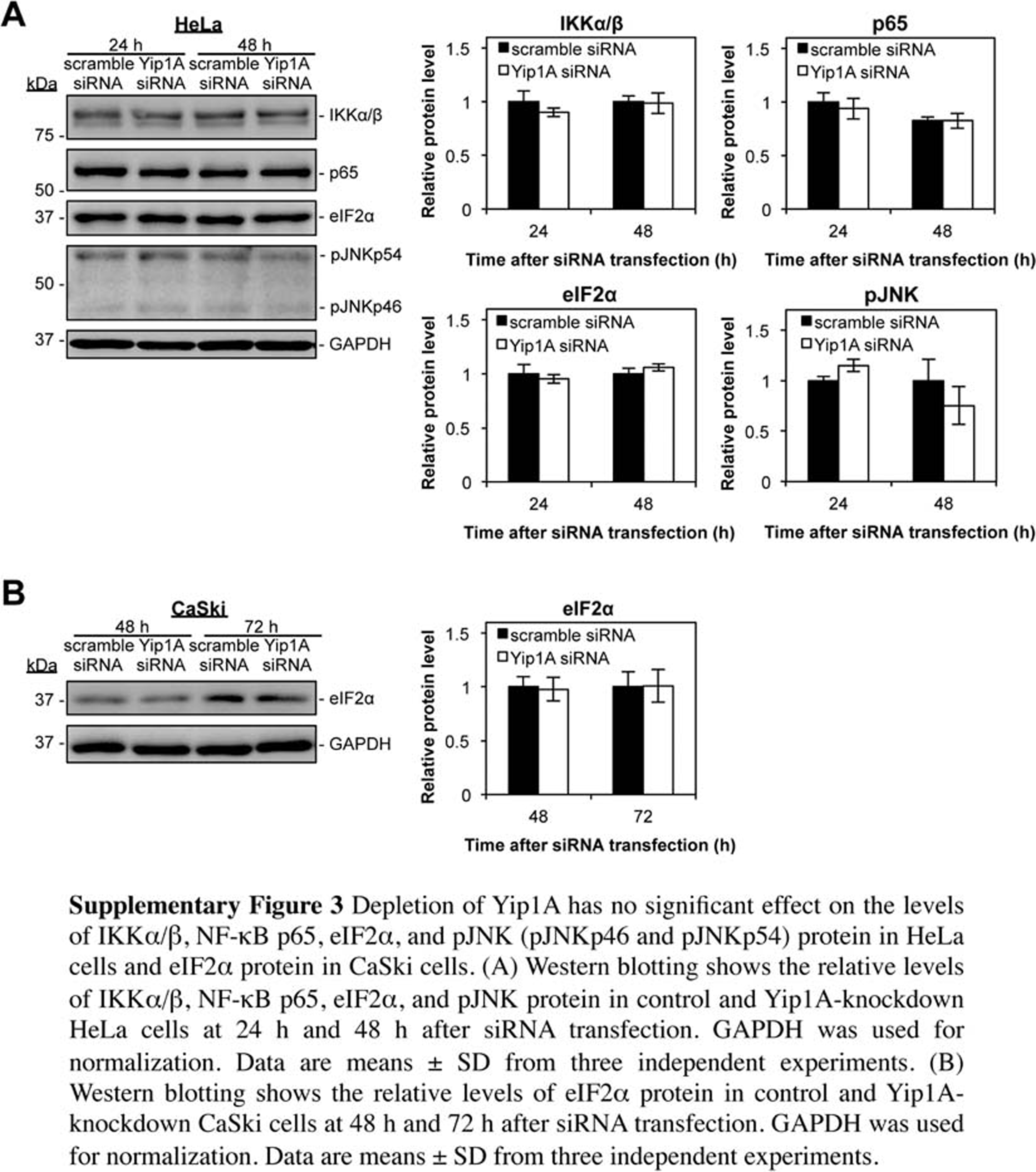

Supplement: Supplementary Figure 3 [file cddis2017147x3.tif]

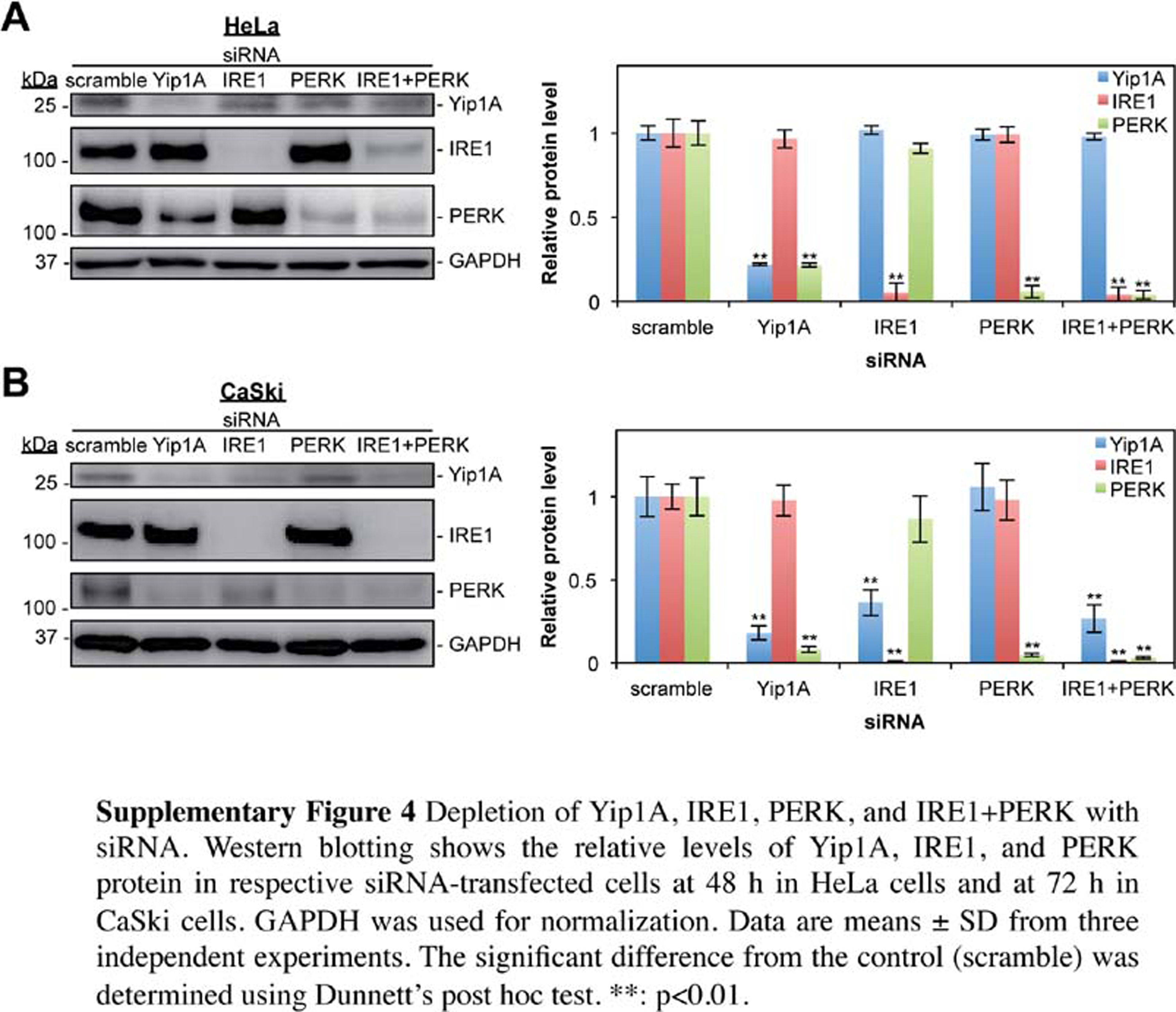

Supplement: Supplementary Figure 4 [file cddis2017147x4.tif]
